# Visual performance of Acrysof ReSTOR compared with a monofocal intraocular lens following implantation in cataract surgery

**DOI:** 10.3892/etm.2012.740

**Published:** 2012-10-10

**Authors:** JING JI, XIAOLIN HUANG, XIANQUN FAN, MIN LUO

**Affiliations:** Department of Ophthalmology, Shanghai Ninth Hospital, Shanghai JiaoTong University School of Medicine, Shanghai, P.R. China

**Keywords:** contrast sensitivity, wavefront analysis, multifocal intraocular lens, pseudoaccommodation, visual performance

## Abstract

The aim of this study was to compare the visual performance of Acrysof ReSTOR and Acrysof Natural intraocular lenses (IOLs) following cataract surgery. A randomized prospective study was performed in which 64 eyes (51 patients) were divided randomly into two groups. Monofocal IOLs (Acrysof Natural) were implanted into 34 eyes (27 patients) and multifocal IOLs (Acrysof ReSTOR) were implanted into 30 eyes (24 patients) using phacoemulsification surgery. The corrected distance visual acuity, near visual acuity, pseudoaccommodation, contrast sensitivity (CS) and wavefront analysis were measured at 1 week, 1 month and 3 months after surgery. The distance vision of the monofocal and ReSTOR patients improved equally with glasses (P<0.05). A greater improvement in near vision without glasses was observed in the ReSTOR-implanted patients (P<0.01). The CS values of the multifocal IOL group were significantly lower than those of the monofocal IOL group for all spatial frequencies tested (P<0.05). The spherical aberration was significantly higher in the multifocal IOL group compared with the monofocal IOL group (P<0.05). We observed no differences in coma between the two groups. The difference in the amplitude of pseudoaccommodation between the two groups was statistically significant (−3.14±0.91 D in the ReSTOR group vs. −1.03±0.33 D in the Natural group, P<0.01). The improvement in near vision was significantly more evident in the ReSTOR patients. Compared with the monofocal IOL lens, the multifocal lens is able to increase the amplitude of pseudoaccommodation. However, increased spherical aberration may contribute to lower CS values in the multifocal IOL group.

## Introduction

Intraocular lenses (IOLs) are designed to provide the best quality of vision following cataract surgery. Monofocal IOLs are capable of providing excellent distance vision quality. However, patients with monofocal IOLs require glasses for near vision. Multifocal IOLs have been developed to reduce the patients’ dependence on glasses. Clinically, multifocal IOLs have been reported to provide functional near distance vision with an acceptable level of satisfaction (1). Snellen visual acuity insufficiently describes the quality of eye optics before and after surgery (2). The deficiencies in the optical quality of vision may be effectively evaluated using a contrast sensitivity (CS) test and wavefront analysis. Improvements in uncorrected near visual acuity have been achieved with multifocal IOLs but a loss of clarity, low CS and complaints of halos and glare have also been reported (3). Yoon *et al*(4) reported spherical aberration to be one of the most significant higher-order aberrations (HOAs) that reduce retinal image quality. The present study compared the improvement in near vision and distance vision-associated limitations between ReSTOR and monofocal IOLs following their implantation using phacoemulsification. In addition, the pseudoaccommodation, CS and HOA values of the patients were measured.

## Patients and methods

### Patients

This randomized study was conducted in the Ophthalmology Department of the Shanghai Ninth People’s Hospital (Shanghai, China). Patients between 50 and 75 years old with age-associated cataracts were enrolled. Patients with ocular diseases, including corneal astigmatism of >1.5 diopters, glaucoma, retinal abnormalities and surgical complications were excluded. Out of 64 eligible eyes (51 patients), 30 (24 patients) were randomly assigned to the ReSTOR group and 34 (27 patients) to the Acrysoft Natural group. The ReSTOR IOLs were multifocal and Acrysoft Natural IOLs were monofocal.

### Surgery

All surgery was performed by one experienced surgeon between January 2009 and December 2011 using the standard surgical technique involving retrobulbar anesthesia by 2% lidocaine, a 3.0-mm scleral tunnel incision on the steepest meridian, phaco-chop, irrigation/aspiration of cortical material, IOL implantation in the capsular bag using the injector system and no suture. Phacoemulsification was performed using an Infiniti (Alcon, Fort Worth, TX, USA) device.

### Follow-up

A combination of antibiotic and steroid eyedrops (TOBRADEX^®^ sterile ophthalmic suspension) and 0.1% diclofenac sodium was administered to the patients postoperatively, initially 4 times per day and then less frequently over a 14 day period. The patients were followed up at 7, 30 and 90 days after surgery. Refraction, best spectacle-corrected distance visual acuity (BCDVA), near visual acuity (UNVA) with glasses or without glasses, pseudoaccommodation, slit-lamp examination, fundoscopy, aberrometry (total) and CS were evaluated. A monocular high-contrast Snellen visual assessment for distance was performed using the early treatment diabetic retinopathy study chart with BCDVA at 4 m under photopic conditions. Near visual acuities were measured using the Rosenbaum near-vision card at a distance of 33 cm. Pseudoaccommodation was measured with a coincidence refractometer. Minor eyeglasses were then increased according to a range of −0.25 D until the patients reported that their vision was not clear. Pseudoaccommodation = 2.50 - near added value + the absolute refractive value of minor eyeglasses. CS was tested using a CGT-1000 contrast sensitivity testing instrument (Takagi, Japan). The test measured the spatial frequencies at 0.7, 1.0, 1.6, 2.5, 4.0 and 6.3 degrees. All measurements recorded under photopic conditions were performed under 80 and 5 cd/m^2^ mesopic conditions. Wavefront analysis was performed with an Allegretto Wavelight Analyzer (Wavelight Technologe Inc., Erlayen AG). The Hartmann-Shack method was used to measure the root mean square (RMS) of coma $\left({\text{Z}}_{3}^{1}\right)$, spherical aberration $\left({\text{Z}}_{4}^{0}\right)$ and high-order aberration $\left({\text{Z}}_{3}^{-1}\right)$ with 4.0 and 6.0 mm pupils. All the measurements for CS and HOAs were obtained using the best spectacle-corrected acuity.

### Statistical analysis

Statistical analysis was performed using the mean and standard deviation for quantitative variables. The comparison of quantitative variables was performed using analysis of variance (ANOVA) and the differences were calculated using a multiple comparison Tukey’s test. For multiple measurements, Bonferroni correction was applied when necessary. All results were presented with 95% confidence limits. P<0.05 was considered to indicate statistically significant differences.

## Results

### Patients

A total of 64 eyes (51 patients) were enrolled in the study. There were 10 (41.7%) males and 14 (58.3%) females in the ReSTOR group with a mean age of 63.01±9.08 (range, 52–71) years. There were 12 (44.4%) males and 15 (55.6%) females in the Natural group with a mean age of 63.15±8.90 (range, 55–75) years.

### Visual acuity analysis

On postoperative day 7, 40% (12/30) of patients achieved a BCDVA ≥0.5 in the ReSTOR group and 71% (24/34) did so in the Natural group. The percentage of patients with an uncorrected UNVA ≥0.3 was 33% (10/30) in the ReSTOR group and 5.9% (2/34) in the Natural group. On postoperative day 30, the percentage of patients with a BCDVA ≥0.5 was 86.7% (26/30) in the ReSTOR group and 85% (29/34) in the Natural group. The percentages of patients with a UNVA ≥0.3 were 63% (19/30) and 8.8% (3/34) in the ReSTOR and Natural groups, respectively. On postoperative day 90, the percentage of patients with a BCDVA of ≥0.5 was 93.3% (28/30) in the ReSTOR group and 73.5% (25/34) in the Natural group. The percentages of patients with UNVAs ≥0.3 were 86.7% (26/30) and 11.8% (4/34) in the ReSTOR and Natural groups, respectively (Table I).

The mean BCDVA values in the ReSTOR and Natural groups were: 0.59±0.11 and 0.61±0.09, respectively, on postoperative day 7 (P=0.83); 0.64±0.17 and 0.74±0.16, respectively, on postoperative day 30 (P=0.33); and 0.71±0.18 and 0.75±018, respectively, on postoperative day 90 (P=0.77). No significant differences were observed in BCDVAs between the ReSTOR and Natural groups. (P>0.05; Table II).

The mean UNVA values in the ReSTOR and Natural groups were: 0.29±0.07 and 0.18±0.08, respectively, on postoperative day 7; 0.48±0.09 and 0.24±0.06, respectively, on postoperative day 30 (P=0.15); and 0.58±0.09 and 0.21±0.16, respectively, on postoperative day 90 (P=0.008). No significant difference was observed between the two groups on postoperative days 7 and 30 (P>0.05). A significant difference was observed in UNVAs between the two groups on postoperative day 90 (P<0.05). All the average BCDVAs were obtained from Snellen visual chart values and the UNVAs were obtained using Jaeger visual charts (Table III).

### CS analysis

The results of CS testing are shown in Table IV. On postoperative days 7, 30 and 90 the patients with ReSTOR IOLs exhibited lower CS values than their counterparts with Acrysof Natural IOLs under mesopic and photopic conditions. Statistically significant differences were observed in all spatial frequencies between the groups at all follow-up times (P<0.05).

### Wavefront analysis

Wavefront analysis at each postoperative follow-up, including the RMS of the total aberration for 4.0-mm and 6.0-mm pupils in each group, is shown in Table V. ${\text{Z}}_{4}^{0}$ was significantly higher in the multifocal IOL group than in the monofocal IOL group (P<0.05). No differences were observed in ${\text{Z}}_{3}^{1}$ values between the two groups.

The difference in the amplitude of pseudoaccommodation between the two groups was statistically significant (−3.14±0.91 D in the ReSTOR group vs. −1.03±0.33 D in the Natural group, P<0.01). There were no differences observed in ${\text{Z}}_{3}^{-1}$ and ${\text{Z}}_{3}^{1}$ values between the two groups in 4.0-mm pupils (P>0.05). In the Natural or ReSTOR group, RMS, ${\text{Z}}_{3}^{-1}$, ${\text{Z}}_{3}^{1}$ and ${\text{Z}}_{4}^{0}$ values exhibited significant differences between 4.0 mm and 6.0-mm pupils. The values increased in the 6-mm pupil.

## Discussion

One of the major defects of monofocal IOLs as replacements for human crystalline lenses is the fixed focus of the IOLs (5). Although patients may see well at a distance following cataract surgery, reading spectacles are generally required for near vision. To address this issue, multifocal IOLs that provide refractive correction for near and distance vision are now available (6). Brydon *et al*(7) compared the BCDVA, UBVA, UNVA and BCNVA values of patients with multifocal or monofocal IOLs. The results demonstrated that there was no difference between the two groups in terms of BCDVA and UNVA values. However, patients with multifocal IOLs may have improved near visual acuity without glasses. In the present study, the number of patients in the ReSTOR group with a UNVA of 0.3 or more was larger than that of the Natural group during all the follow-up periods. However, there was no difference between the two groups in terms of the number of patients with a BCDVA of 0.5 or more. These results indicate that the multifocal IOLs may decrease the dependence on glasses of patients who attained satisfactory distance visual acuities.

Accommodation is defined as the eye’s ability to focus on near objects by changing its refractive power (8). As the human lens ages, its accommodative amplitude decreases. People cannot obtain clear visual acuity when they see things nearby. This phenomenon is called presbyopia. Patients with an implanted pseudophakic eye are similar to an absolute presbyope following cataract surgery. A ReSTOR IOL employs a central apodized diffractive zone surrounded by a purely refractive outer zone. It has a central 3.6-mm diffractive optic region where 12 concentric diffractive zones on the anterior surface of the lens divide light into two diffraction orders to create two lens powers. The central 3.6-mm zone is surrounded by a region that has no diffractive structure over the remainder of the 6-mm diameter lens. The near correction is calculated at +4.0 D at the lens plane, resulting in approximately +3.2 D at the spectacle plane. This provides 6 D of pseudoaccommodation. Sugitani, Hardman *et al* and Nakazawa and Ohtsuki (9–11) reported that the pseudophakic eye also reserves 2 D of amplitude for pseudoaccommodation. In the present study, the pseudoaccommodation in the ReSTOR group approaches 4 D, which allows the patient to achieve complete distance vision. Additionally, patients in the Natural group exhibited some pseudoaccommodation. However, the difference between the two groups was not significant.

Although the multifocal IOLs may afford cataract patients complete distance visual acuity, they may increase the depth of focus in exchange for a loss of CS. It has been reported that AMO Array multifocal IOLs [Tecnis ZM900,Abbott Medical Optics, Inc. (AMO)] may cause more glare and lower CS than monofocal IOLs (12). Bellucci (13) reported that patient satisfaction was no higher for multifocal IOLs than monofocal IOLs and the poor visual performance was attributed to the reduction of CS and the presence of halos. The present study demonstrated that CS values were decreased and HOAs were increased in the multifocal IOL group. CS values were significantly lower in the ReSTOR group compared with the Natural group for all spatial frequencies under all conditions. HOAs, particularly spherical aberrations, were increased significantly in the ReSTOR group. The present study revealed that there may be an association between decreased CSs and increased spherical aberrations in the ReSTOR group. A possible cause of this issue is the division of light energy through the two focal points produced by the multifocal IOL, suggesting that spherical aberration is involved in decreasing the CS of multifocal IOL-implanted eyes. Residual refractive errors and delicate decentration of the IOLs associated with the pupil size may affect the visual performance of the patients. Thus, spherical aberration increases in multifocal IOLs with large pupil sizes. The present study revealed that the value of the wavefront increased with the 6-mm pupil size for RMS, ${\text{Z}}_{3}^{-1}$ and ${\text{Z}}_{4}^{0}$ at all follow-up times. This suggests that visual acuity decreases when patients with multifocal IOLs drive at night.

In conclusion, ReSTOR IOLs provide the additional benefit of uncorrected near vision. ReSTOR also provides patients with a comfortable distance vision that is comparable to that of monofocal IOLs and a comfortable near vision without glasses that is significantly superior to that of monofocal IOLs. However, decreased CSs are associated with low visual performance satisfaction in multifocal IOL-implanted patients and spherical aberration appears to be a key contributor to reduced CS in these patients. Thus, when doctors select the type of IOL for cataract patients, the patients’ personal requirements should be taken into account in order to improve the quality of their lives.

## Figures and Tables

**Table I:** Post-operative BCDVA and UNVA.

Follow-up time (days)	Visual acuity	ReSTOR group (%)	Natural group (%)
7	BCDVA ≥0.5	12 (40)	24 (71)
	UNVA ≥0.3	10 (33)	2 (5.9)
30	BCDVA ≥0.5	26 (86.7)	29 (85)
	UNVA ≥0.3	19 (63)	3 (8.8)
90	BCDVA ≥0.5	28 (93.3)	25 (73.5)
	UNVA ≥0.3	26 (86.7)	4 (11.8)

BCDVA, best spectacle-corrected distance visual acuity; UNVA, near visual acuity.

**Table II:** Postoperative BCDVA.

	BCDVA		
Characteristic	Postoperative day 7	Postoperative day 30	Postoperative day 90
ReSTOR (mean ± SD)	0.59±0.11	0.64±0.17	0.71±0.18
Natural (mean ± SD)	0.61±0.09	0.74±0.16	0.75±0.18
P-value	0.83	0.33	0.77

Decimal data supplied. BCDVA, best spectacle-corrected distance visual acuity.

**Table III:** Postoperative UNVA.

	UNVA		
Characteristic	Postoperative day 7	Postoperative day 30	Postoperative day 90
ReSTOR (mean ± SD)	0.29±0.07	0.48±0.09	0.58±0.09
NATURAL (mean ± SD)	0.18±0.08	0.24±0.06	0.21±0.16
P-value	0.56	0.15	0.008

Decimal data supplied. UNVA, near visual acuity.

**Table IV:** Contrast sensitivy.

	Mesopic			Photopic		
Degree (spatial frequency)	ReSTOR (mean ± SD)	Natural (mean ± SD)	P-value	ReSTOR (mean ± SD)	Natural (mean ± SD)	P-value
Postoperative day 7						
6.3	21.00±7.32	46.83±9.25	0.02	29.10±11.2	45.88±22.14	0.03
4.0	14.27±9.43	34.26±2.45	0.04	24.8±11.86	47.96±11.87	0.02
2.5	14.45±5.12	29.80±1.8	0.03	18.5±8.66	35.03±8.68	0.02
1.6	9.80±10.83	22.45±1.24	0.02	12.14±20.65	29.40±4.87	0.02
1.0	4.86±11.81	15.98±11.93	0.00	5.39±21.2	15.00±11.17	0.00
0.7	3.78±8.3	11.27±5.9	0.02	2.4±1.9	3.70±11.62	0.02
Postoperative day 30						
6.3	20.41±2.4	44.33±2.17	0.037	31.49±3.72	43.11±2.52	0.04
4.0	16.3±3.91	37.77±6.93	0.038	30.7±2.91	41.57±5.1	0.02
2.5	13.37±8.16	33.91±11.13	0.00	19.94±9.1	30.66±9.5	0.01
1.6	9.88±1.37	19.69±7.71	0.01	9.98±2.11	17.50±4.23	0.04
1.0	7.26±10.59	15.0±3.20	0.03	7.31±6.09	13.0±1.66	0.03
0.7	1.0±0.2	2.20±1.12	0.04	3.87±5.87	1.06±0.33	0.02
Postoperative day 90						
6.3	40.83±2.5	66.35±2.61	0.02	33.46±2.86	40.77±7.71	0.02
4.0	47.28±2.68	52.96±2.63	0.01	15.42±7.35	22.69±8.99	0.03
2.5	33.46±2.55	41.67±2.07	0.03	15.57±7.22	22.83±9.24	0.02
1.6	16.66±7.01	28.69±13.39	0.01	8.70±16.44	17.19±5.41	0.01
1.0	8.16±6.39	16.84±5.58	0.02	4.8±1.03	8.14±4.08	0.04
0.7	3.66±1.88	9.68±2.34	0.00	2.76±7.90	8.86±4.35	0.04

**Table V:** Wavefront analysis.

	4.0-mm pupil			6.0-mm pupil		
Aberrations	ReSTOR (μm)	Natural (μm)	P-value	ReSTOR (μm)	Natural (μm)	P-value
Postoperative day 7^a^						
RMS	0.31±0.15	0.547±1.01	0.008^d^	0.39±0.23	1.00±0.55	0.00^d^
${\text{Z}}_{3}^{-1}$	−0.0057±0.11	0.0568±0.42	0.06	−0.018±0.20	0.271±0.53	0.02^d^
${\text{Z}}_{3}^{1}$	−0.0243±0.21	0.041±0.31	0.15	0.03±0.144	0.04±0.08	0.08
${\text{Z}}_{4}^{0}$	0.003±0.08	0.070±0.31	0.01^d^	0.04±0.104	0.17±0.52	0.03^d^
Postoperative day 30^b^						
RMS	0.211±0.109	0.623±0.18	0.01^d^	0.39±0.03	1.00±0.39	0.00^d^
${\text{Z}}_{3}^{-1}$	0.021±0.15	0.022±0.55	0.51	−0.08±0.17	0.18±0.51	0.04^d^
${\text{Z}}_{3}^{1}$	−0.011±0.135	−0.014±0.58	0.32	0.09±0.15	0.12±0.03	0.02
${\text{Z}}_{4}^{0}$	0.024±0.07	0.077±0.20	0.05^d^	0.014±0.09	0.19±0.49	0.01^d^
Postoperative day 90^c^						
RMS	0.21±0.1	0.50±0.21	0.00^d^	0.41±0.28	0.96±0.31	0.02^d^
${\text{Z}}_{3}^{-1}$	0.053±0.19	0.10±0.37	0.22	−0.05±0.35	−0.26±0.59	0.019^d^
${\text{Z}}_{3}^{1}$	0.03±0.15	0.08±0.15	0.10	0.028±0.2	0.05±0.04	0.02
${\text{Z}}_{4}^{0}$	0.05±0.13	0.10±0.21	0.00^d^	0.03±0.10	0.14±0.14	0.00^d^

Pseudoaccommodation: ReSTOR group −3.14±0.91 D, Natural group −1.03±0.33 D, P<0.01.

a Data comparison between two sizes of pupil, P=0.015.

b Data comparison between two sizes of pupil, P=0.0261.

c Data comparison between two sizes of pupil, P=0.001. RMS, root mean square.

## References

[1] Javitt JC, Steinert RF (2000). Cataract extraction with multifocal intraocular lens implantation: a multinational clinical trial evaluating clinical, functional, and quality-of-life outcomes. Ophthalmology.

[2] Elliott DB (1993). Evaluating visual function in cataract. Optom Vision Sci.

[3] Dick HB, Krummenauer F, Schwenn O, Krist R, Pfeiffer N (1999). Objective and subjective evaluation of photic phenemena after monofocal and multifocal intraocular lens implantation. Ophthamology.

[4] Yoon G, Macrae S, Williams DR, Cox IG (2005). Causes of spherical aberration induced by laser refractive surgery. J Cataract Refract Surg.

[5] Zhao YE, Li JH, Zhu J, Wang DD, Wang QM (2009). Evaluation of long term visual performance following AcrySof ReSTOR lens implantation. Chin Med J (Engl).

[6] Min Luo, Ji J, Zhao C, Fan X (2010). Clinical study of Acrysof IQ aspheric intraocular lenses. Clin Experiment Ophthalmol.

[7] Brydon KW, Tokarewicz AC, Nichols BD (2000). AMO arry multifocal lens versus monfocal correction in cataract surgury. J Cataract Refract Surg.

[8] Glasser A, Campbell MC (1998). Presbyopia and the optical changes in the human crystalline lens with age. Vision Res.

[9] Sugitani Y (1979). Apparent accommodation (pseudoaccomodation) on pseudophakia. Folia Ophthalmol Jpn.

[10] Hardman Lea SJ, Rubinstein MP, Snead MP, Haworth SM (1990). Pseudophakic accommodation? A study of the stability of capsular bag supported, one piece, rigid tripod or soft flexible implants. Br J Ophthalmol.

[11] Nakazawa M, Ohtsuki K (1983). Apparent accommodation in pseudophakic eyes after implantation of posterior chamber intraocular lenses. Am J Ophthalmol.

[12] de Vries NE, Webers CA, Verbakel F, de Brabander J, Berendschot TT, Cheng YY, Doors M, Nuijts RM (2010). Visual outcome and patient satisfaction after multifocal intraocular lens implantation: aspheric versus spherical design. J Cataract Refract Surg.

[13] Bellucci R (2005). Multifocal intraocular lenses. Curr Opin Ophthalmol.

